# Distinctive Gut Microbiota Is Associated with Diarrheagenic *Escherichia coli* Infections in Chilean Children

**DOI:** 10.3389/fcimb.2017.00424

**Published:** 2017-10-12

**Authors:** Pablo Gallardo, Mariana Izquierdo, Roberto M. Vidal, Nayaret Chamorro-Veloso, Ramon Rosselló-Móra, Miguel O'Ryan, Mauricio J. Farfán

**Affiliations:** ^1^Departamento de Pediatría y Cirugía Infantil, Facultad de Medicina, Campus Oriente-Hospital Dr. Luis Calvo Mackenna, Universidad de Chile, Santiago, Chile; ^2^Programa de Microbiología y Micología, Facultad de Medicina, Instituto de Ciencias Biomédicas, Universidad de Chile, Santiago, Chile; ^3^Grupo de Microbiología Marina, IMEDEA (CSIC-UIB), España, Spain; ^4^Facultad de Medicina, Instituto Milenio de Inmunología e Inmunoterapia, Universidad de Chile, Santiago, Chile; ^5^Laboratorio de Biología Molecular, Clínica Las Condes, Santiago, Chile

**Keywords:** diarrheagenic *Escherichia coli*, diarrhea, indicative species, operational phylogenetic units, OPU, taxa assignment, microbiota

## Abstract

**Background:** Diarrheagenic *Escherichia coli* (DEC) strains are a major cause of diarrhea in children under 5 years of age worldwide. DEC pathogenicity relies on the interaction of bacteria with environmental factors, including the host's resident gut microbiota. Previous reports have shown changes in the gut microbiota's composition during episodes of diarrhea, which may increase the pathogenicity of DEC strains. More intense and detailed identification of microbiota strains specifically associated with DEC infections and disease is needed to pinpoint their role in DEC pathogenicity.

**Aim:** To identify resident indicative bacterial taxa in DEC-positive diarrhea stool samples of Chilean children.

**Methods:** We analyzed 63 diarrheal stool samples from children 1–5 years of age by FilmArray® GI in order to identify a potential pathogen and to group diarrhea episodes into those caused by DEC as sole pathogen (DEC group, 32 samples) and those caused by an enteric virus as sole pathogen (viral group, 31 samples). In addition, 30 stool samples from healthy children, negative for enteric pathogens, were evaluated (healthy group). The 16S rRNA gene was amplified and sequenced using 454 pyrosequencing. Sequences were clustered into operational taxonomic units (OTUs) at 99% identity and their representatives were used to assign them to operational phylogenetic units (OPUs) using a phylogenetic inference approach.

**Results:** Taxa assignment using the OPU approach resulted in a lower number of units but with higher accuracy compared to the OTU approach. Data analysis indicated an increase in sequences belonging to the phylum *Proteobacteria* in the DEC group compared to the viral and healthy groups. Samples displayed a statistically different community structure by sample grouping by redundancy analysis and ANOVA. *Escherichia albertii* (*p* = 0.001), *Citrobacter werkmanii* (*p* = 0.001), *Yersinia enterocolitica*, subsp. *paleartica* (*p* = 0.048), and *Haemophilus sputorum* (*p* = 0.028) were indicative species for the DEC group as compared to the viral and healthy groups.

**Conclusion:** Gut microbiota in Chilean children with DEC-positive diarrhea differed from microbiota associated with enteric virus and healthy children. Indicative species found in this study may prove relevant in advancing our understanding of the relationship between resident gut microbiota and DEC leading to the occurrence of disease.

## Introduction

According to the World Health Organization (WHO), there are nearly 1.7 billion cases of diarrheal disease every year, and 760,000 deaths, representing the second cause of death in children under 5 years of age (World Health Organization, 2014). Diarrheagenic *Escherichia coli* (DEC) are the most common bacterial cause in both developing and industrialized regions, primarily affecting children under 5 years of age (Gomes et al., 2016). DEC infection prevalence has been underestimated, largely due to the lack of surveillance studies and the limited availability of molecular diagnostic techniques. Implementation of molecular techniques allowing simultaneous detection of multiple enteric pathogens, including DEC pathotypes, have shown that the prevalence of these pathotypes in diarrheal infections is higher than previously thought (Buss et al., 2015; Farfán et al., 2016).

DEC strains comprise a group of *E. coli* strains that have acquired several genes conferring the ability to cause a broad spectrum of infections (Nataro and Kaper, 1998). According to the detection of the virulence factors, six classical categories or pathotypes have been described: enteropathogenic *E. coli* (EPEC), Shiga toxin-producing *E. coli* (STEC), enterotoxigenic *E. coli* (ETEC), enteroaggregative *E. coli* (EAEC), enteroinvasive *E. coli* (EIEC), and diffusely adherent *E. coli* (DAEC). In the last years, new *E. coli* pathotypes have emerged as a consequence of a unique combination of virulence traits (Rasko et al., 2011). In general, DEC infection involves three steps: (i) adherence and colonization of the intestinal surface, (ii) production and secretion of virulence factors, and (iii) diarrhea and inflammation (Nataro et al., 1996). These steps are tightly regulated, either by environmental conditions or bacterial regulators that induce or silence the expression of virulence factors (reviewed in Gomes et al., 2016). The expression of specific virulence genes and the ability to efficiently utilize nutrient in the intestinal milieu are key events for the successfully colonization of DEC to intestinal epithelia. However, this phenomenon is not completely understood for DEC, as most studies have focused on molecular mechanism occurring inside the bacteria, with only a few evaluating environmental factors, such as, gut microbiota (Pacheco and Sperandio, 2015; Singh et al., 2015). In the last years, several groups have focused on the role components of the gut microbiota and its association with diarrheal disease caused by enteric pathogens, including DEC strains (Nelson et al., 2012; Curtis et al., 2014; Iversen et al., 2015; Lindsay et al., 2015).

The gut microbiota is proposed to play an important role in human health by acting as a barrier against pathogens, stimulating metabolic function and promoting the development of the immune system (Hooper et al., 2012). Conversely, recent observations strongly suggest that diarrheal infections caused by DEC induces changes in the intestinal bacteria microbiota, and that specific components of gut microbiota can modulate the expression of virulence genes leading to enhancement of the infectious process (Curtis et al., 2014; Singh et al., 2015). Next Generation Sequencing (NGS) platforms have facilitated the quantitative phylogenetic identification of the microbiota at various taxonomic levels (Fraher et al., 2012). Analysis of 16S rRNA gene sequences based on percent identity, using Operational Taxonomic Units (OTUs) or Operational Phylogenetic Units (OPUs) for taxonomic assignment, has facilitated the identification of known and potentially new microorganisms (Yarza et al., 2014).

The epidemiological relevance of DEC infections unpaired by only a small number of studies evaluating DEC associated gut microbiota lead us to characterize the presence and relative abundance of indicative taxa associated with DEC infection in the intestinal microbiota of children under 5 years of age. The main goal of this study was to identify a distinctive gut microbiota in diarrheal samples positive for DEC pathotypes compared to diarrheal samples positive for enteric virus and stool samples from healthy children.

## Patients and methods

### Stool samples

Diarrheal stool samples were collected between January and December 2016 from children 1–5 years of age, consulting at the outpatient clinic of Clínica Las Condes (Santiago, Chile). Samples were tested by FilmArray® GI upon request of treating physicians. Filmarray® GI is a FDA-cleared qualitative PCR system that integrates sample preparation, amplification, detection and analysis. Filmarray® GI panel detects the following gastrointestinal pathogens: viruses (Adenovirus F40/41; Astrovirus; Norovirus GI/GII; Rotavirus A; and Sapovirus I, II, IV and V), bacteria (*C. jejuni; C. coli; C. upsaliensis; C. difficile; P. shigelloides; Salmonella; Yersinia enterocolitica; V. parahaemolyticus; V. vulnificus; V. cholera; Shigella;* and DEC pathotypes EAEC, EPEC, ETEC, STEC, and EIEC) and parasites (*Cryptosporidium; C. cayetanensis; E. histolytica;* and *G. lamblia*).

Stool samples from healthy children were obtained from a cohort residing in the city of Colina (Santiago, Chile), participating in a prospective study for *Helicobacter pylori* infection (O'Ryan et al., 2013). *Helicobacter pylori*-negative stool samples were tested by FilmArray® GI and pathogen-free samples were used in this study. A unique code number was assigned to each sample. Diarrheal samples were grouped into a DEC group (samples only positive for at least one DEC pathotype) or enteric viral group (sample positive only for at least one of the enteric viral pathogens). This study was approved by Clínica Las Condes and Universidad de Chile's ethical committees. Informed consent was not required and samples were anonymized, only retaining age.

### DNA extraction, PCR amplification, and pyrosequencing

Total DNA was extracted from each stool using the QIAamp Fast DNA Stool Mini kit (Qiagen), quantified using a Synergy HT® spectrophotometer (Biotek™) and stored at −20°C until PCR amplification. DNA was amplified using a 2-step process. First, the 16S rRNA gene was amplified using the primers GM3 and 1492R, then a nested PCR was performed using the GM3-PS forward primer and a different 907-PS reverse primer for each sample in a 7-cycle reaction. Amplicons were purified and the concentration of the purified product was determined. Equimolar mixtures of the amplicons (10–12 samples each) were shipped to Macrogen Inc. (Seoul, Korea) for pyrosequencing through 454 GS-FLX using 1/8 plate for each 10-sample mixture (Pooled samples). Primer sequences are listed in Supplementary Table 1.

### Sequence trimming and operational taxonomic unit (OTU) clustering

Data was processed and trimmed using the Mothur pipeline for 454 pyrosequencing. Sequences were grouped according the 907-PS primer barcode in each pool, and low-quality sequences were removed. Low quality sequences were defined as sequences under 300 bp, with a window size and average quality score of 25, a maximum homopolymer of 8 nucleotides, without ambiguities and reading mismatches with barcodes primers. Sequences were clustered into OTUs at 99% identity using the MacQiiME software; chimeras were removed after OTU identification using the same software. The longest sequence from each cluster was selected as the representative OTU sequence. Trimmed sequences and quality files are deposited in the European Nucleotide Archive under accession number ERP103982.

### Phylogenetic tree reconstruction

Representative OTU sequences were aligned using the SILVA Ref 123 database and the SINA alignment tool included in the ARB 6.0 program (Ludwig et al., 2004; Pruesse et al., 2012). Aligned sequences were inserted using the parsimony tool of ARB to the non-redundant SILVA Ref 123 database and the three closest relatives for each sequence were selected. This process was repeated using the LTP 123 database. Closest relatives selected from both the NR SILVA Ref 123 and LTP 123 databases were merged, and a phylogenetic tree was built using the neighbor joining algorithm with the Jukes-Cantor correction and adding the 30% conservational filter to remove hypervariable positions. All alignments were manually reviewed and supervised. For the final tree reconstruction, the closest relative sequences selected with an additional set of about 750 supporting sequences (highest quality in the LTP and covering a balanced representation of all major phyla of both Bacteria and Archaea domains) (Munoz et al., 2016) were used for a neighbor joining reconstruction. The tree was rooted with Archaea from the LTP 123 database, and the branch stability of the tree (named NJ_30) was evaluated using bootstrap analysis as implemented in the ARB software. Aligned OTU sequences were finally inserted into the tree using the ARB parsimony tool.

### Phylogenetic affiliation and operational phylogenetic units (OPU) assignment

OTU sequences were grouped into OPUs based on manual inspection of the NJ_30 tree and monophyletic clustering. OPUs were defined as the smallest monophyletic clade containing one or more amplified sequences affiliating with at least one reference sequence, preferably from a type strain, available in public repositories. When possible, a type sequence from the LTP database was used as the OPU reference, when this was not possible, sequences from the other available databases were used. When an identity value of amplicons with type strain sequences was >98.7%, it was considered to belong to the same species. For identity values <98.7 and >94.5% with the closest relative type strain the amplicons were considered of the same genus but from a different yet unclassified species. When no species or genus was close enough, sequences were clustered into families or classes, using the highest possible identity value (>98%).

### Sample size and statistical analysis

Sample size was determined with the G-Power software (Faul et al., 2007). Considering a type I error of 0.05 and a potency of 80% and effect size of 0.4176, we calculated that a minimal of 60 samples were required, 20 samples per group. Effect size was obtained based on a pilot study done in our laboratory that compared 10 samples of each group defined above. Sequence abundances of OPUs and samples were coded as an entry matrix. For data normalization, absolute read counts were transformed into relative abundances. Rarefaction curves and ß-Diversity were calculated using the “betapart” and “vegan” packages in RStudio software (version 1.0.136). For group comparison, analysis of variance (ANOVA) test followed by Bonferroni's multiple comparisons test using software Prisma 6 were performed. A *p* < 0.01 was considering statistically significant.

Clustering analysis, heatmaps, redundancy data analysis (RDA), and ANOVA were also performed in RStudio. For RDA analyses, data was transformed using a double-square root transformation prior to homogenizing group variances and comparison by ANOVA. RDA was run with a maximum of 999 combinations, in order to determine significant axes. Determination of indicative taxa was performed following De Cáceres and Legendre's protocol of permutations for indicator species analysis by combining site groups (De Cáceres and Legendre, 2009; De Cáceres et al., 2010). The indicator value (IndVal) indicates whether a species was randomly identified as a particular group or site or not. The *p*-value is the proportion of permutations that yielded the association values greater than or equal to those observed for the unpermuted data. Indicative species have a high IndVal (>0.28) and a low *p*-value (<0.05).

## Results

### Taxa assignments using OTUs and OPUs

We analyzed a total of 93 stool samples, 32 diarrheal samples positive for at least one of the five DEC pathotypes (DEC group), 31 diarrheal samples positive only for enteric viruses (viral group), and 30 stool samples from healthy children (healthy group). Table 1 summarizes the microbiological and gene sequencing findings of the stool samples included in this study.

**Table 1:** Overall microbiological and gene sequencing findings in stool samples included in this study.

**Characteristics**	**Groups**		
	**DEC**	**Viral**	**Healthy**
Number of samples	32	31	30
Age (months)^*^	19 [15–37.5]	29 [20–39]	42 [18–50]
Median [Interquartile range]			
Pathogens detected (number of samples)	EAEC (7)	Adenovirus (2)	
	EAEC, EPEC (3)	Adenovirus, Norovirus (1)	
	EIEC (1)	Astrovirus (2)	
	EPEC (14)	Astrovirus, Norovirus (1)	
	ETEC (4)	Norovirus (8)	
	STEC (3)	Norovirus, Sapovirus (2)	
		Rotavirus (9)	
		Sapovirus (6)	
Number of reads	12,014 ± 4,978	12,101 ± 7,850	13,255 ± 4,637
Average ± SD			
Reads after trimming	6,386 ± 3,628	6,435 ± 3,816	7,745 ± 3,116
Average ± SD			
OTUs	174 ± 147	266 ± 198	238 ± 159
Average ± SD			
OPUs	68 ± 33	87 ± 40	101 ± 46
Average ± SD			

* *p = 0.03*.

**Figure 1:**
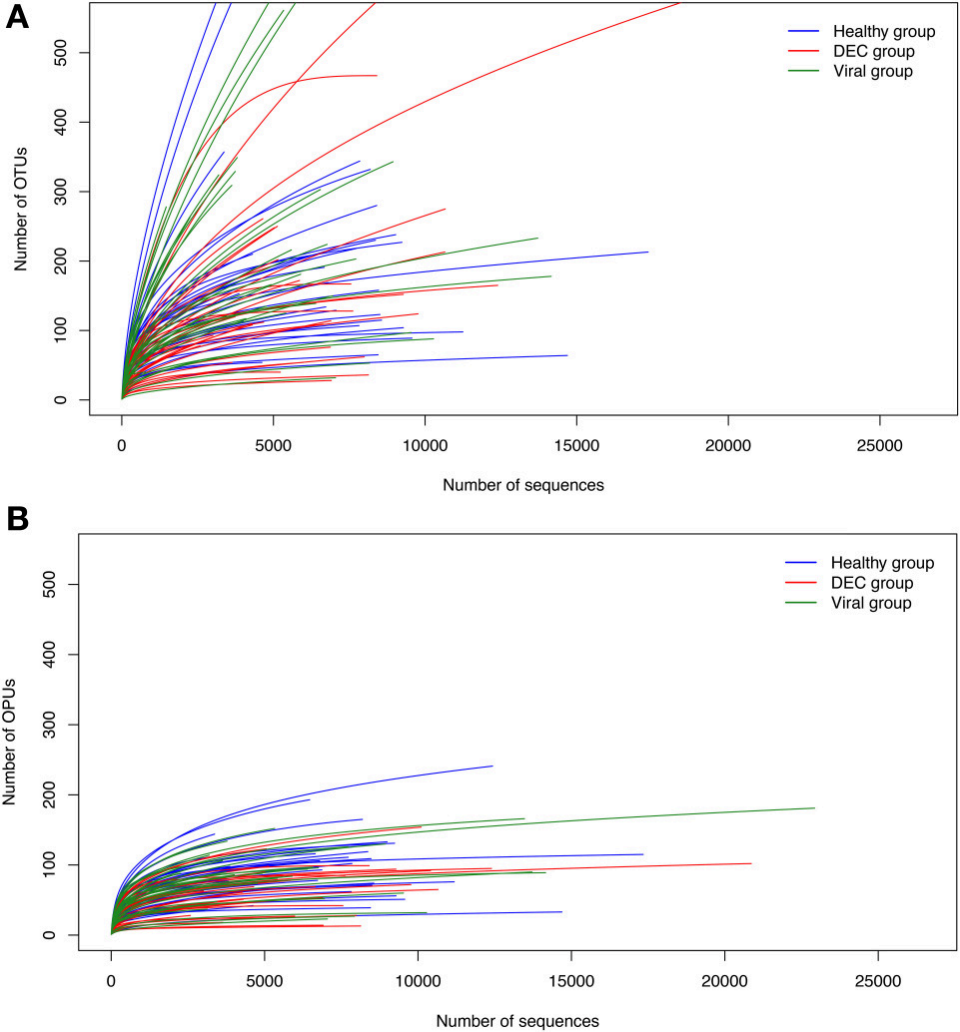
Rarefaction curves for OTU and OPU approaches. Rarefaction curves based on the Operational Taxonomic Units (OTUs) **(A)** and Operational Phylogenetic Units (OPUs) **(B)** detected and their occurrence in DEC (red), viral (green), and healthy (blue) samples. Each line represents an independent sample.

We did not find significant difference in age between all the groups (*p* = 0.03). We identified 20,644 OTUs which were used to build a phylogenetic tree, using the neighbor joining algorithm (Supplementary Figure 1). We identified 852 OPUs, with a mean of 85 OPUs ± 42 per sample. The relationship between the number of OTUs or OPUs and the number of sequences analyzed is shown in Figure 1. OPU curves approached saturation earlier than OTU curves, indicating a potential overestimation of units when using a traditional OTU approach. Group analysis using Jaccard and Sorensen ß-diversity indexes revealed no differences in OPU richness between groups (Supplementary Figure 2).

### Community OPU profile at different taxonomic levels

**Figure 2:**
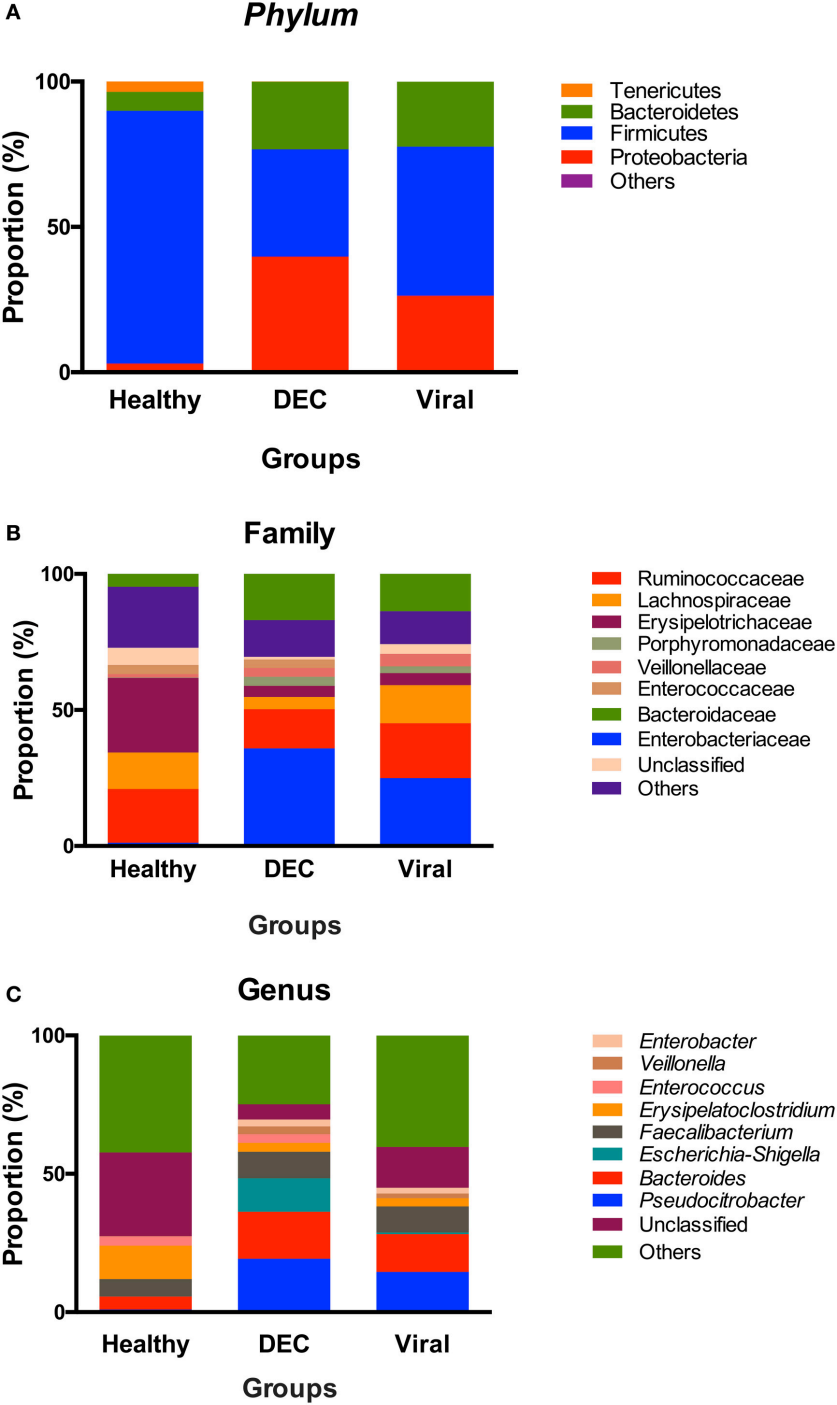
Community profile at different taxonomic levels. Relative abundance of taxa at the level of: **(A)** phyla, **(B)** family, and **(C)** genus. Each color represents a different taxonomic unit. Taxa units were organized based on the 8 most abundant taxa; less representative taxa were grouped as others.

At the phylum level, we observed a lower proportion of taxa belonging to *Firmicutes* in the DEC group compared to the viral (36 vs. 51%, *p* < 0.01) and healthy (36 vs. 86%, *p* < 0.01) groups. Conversely, in the DEC group there was a higher proportion of *Proteobacteria* as compared to the healthy group (40 vs. 3%, *p* < 0.01) and the viral group (40 vs. 26%, *p* < 0.01). Additionally, we detected a higher proportion of *Bacteroidetes* in the DEC group as compared to the healthy group (23 vs. 6%, *p* < 0.01; Figure 2A). At the family level, the most abundant families in the DEC samples were *Enterobacteriaceae, Bacteroidaceae*, and *Ruminococcaceae*. When comparing the three groups, *Enterobacteriaceae* was more abundant in the DEC group as compared to the healthy (36 vs. 1%, *p* < 0.01) and viral (36 vs. 25%, *p* < 0.01) groups (Figure 2B). At the genus level, we found that compared to the healthy group, DEC samples had a greater abundance of *Pseudocitrobacter* (1 vs. 19.3%, *p* < 0.01), *Bacteroides* (4.7 vs. 17%, *p* < 0.01), *Escherichia-Shigella* (0.1 vs. 12%, *p* < 0.01). Compared to the viral group, DEC samples had a greater abundance *Escherichia-Shigella* (0.6 vs. 12%, *p* < 0.01; Figure 2C).

### Differences in OPU composition by abundance and redundancy analyses

Of the 852 OPUs identified, 340 were associated to specific species, 300 to a characterized genus, 58 to more than one species, and 154 to unassigned genus. Species composition and abundance in each group is shown in Supplementary Figure 3.

**Figure 3:**
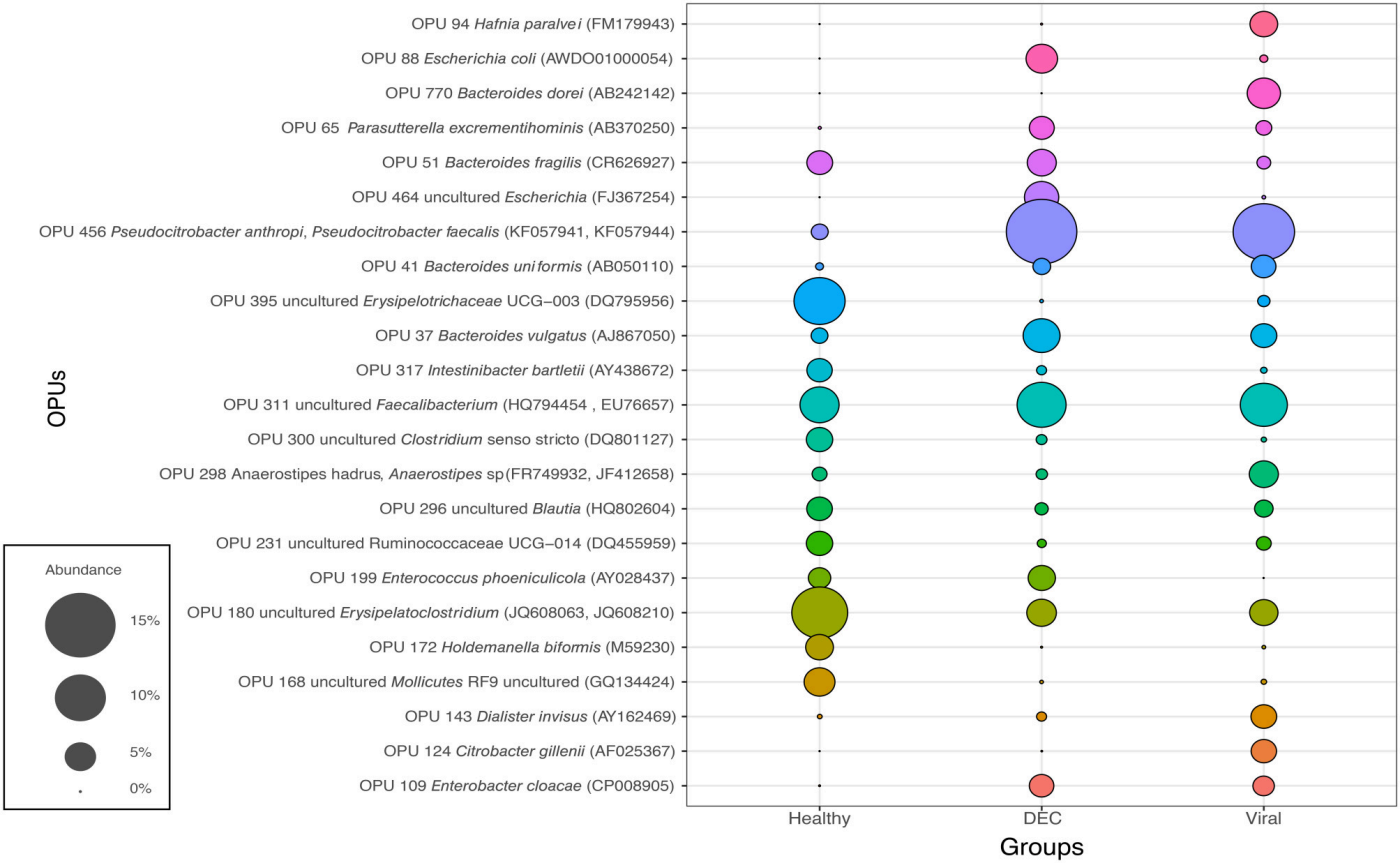
Representation of the 10 most abundant OPUs among groups: Abundance of a total number of 23 OPUs was expressed on percentage (from 0% to 15%). Each color represents an OPU and access number of representative species indicated on parenthesis.

Abundance for each OPU was calculated and the 10 most abundant OPUs of each group was obtained (Figure 3). We identified a total of 23 OPUs within the three groups and 17 OPUs were present in all three groups. In the DEC group, we found that OPUs related to *Escherichia* genus (OPU 88 and OPU 464) were present only in this groups with few exceptions. *Bacteroides dorei* (OPU 770) was the sole species detected only in the viral group. In the healthy group, three taxa were highly abundant compared to the DEC and viral groups (OPU 168, OPU 172, OPU 180, and OPU 395). *Pseudocitrobacter anthropic/faecalis* (OPU 456), *Enterobacter cloaclae* (OPU 109), and *Parasutterella excrementihominis* (OPU 65) were abundantly present in diarrheal samples compared to healthy group.

**Figure 4:**
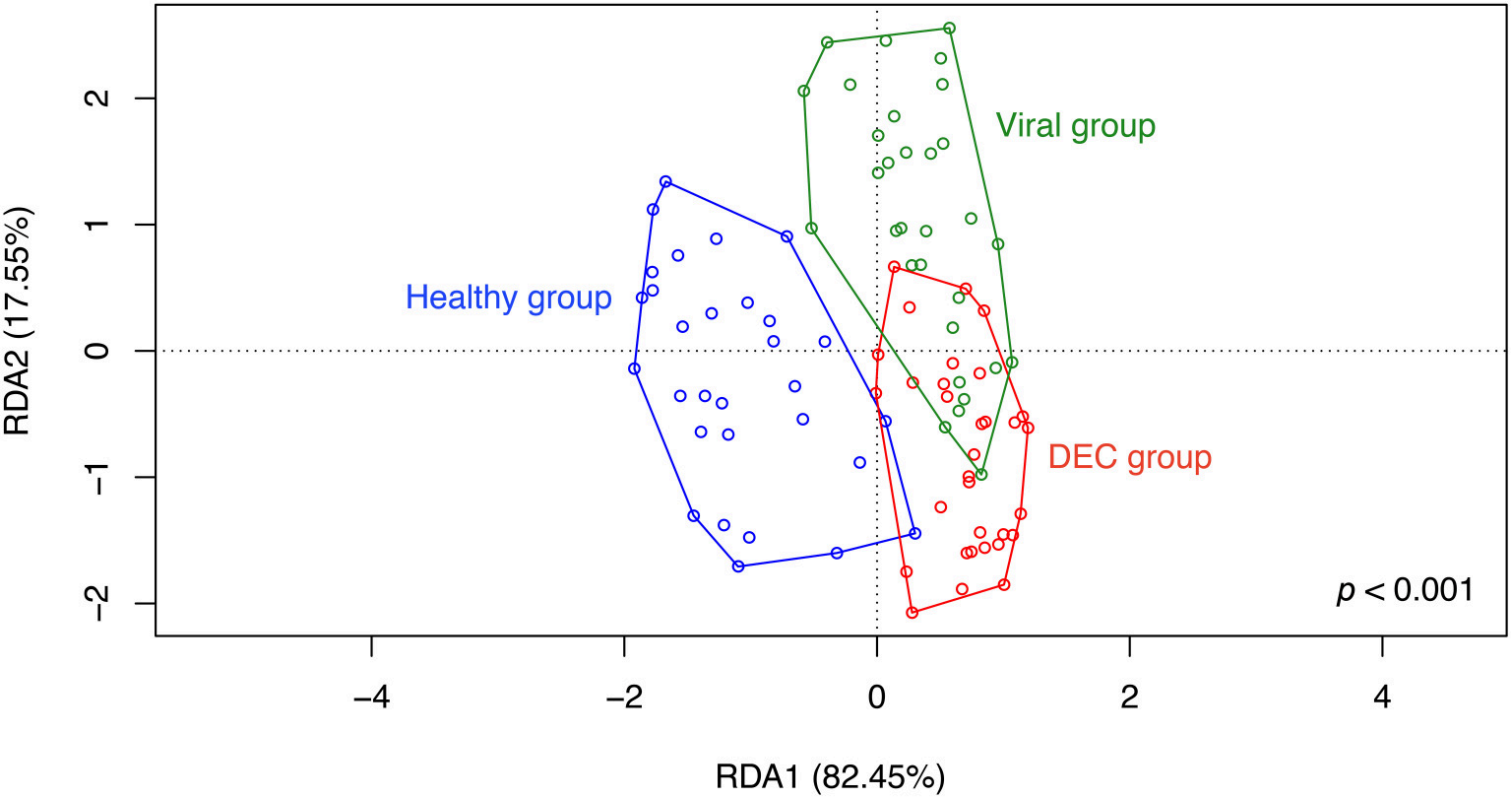
Community structure of groups. Redundancy analysis (RDA) was conducted using sample classification as the explanatory matrix and relative OPU diversity as the response matrix. Data was normalized with a double square root transformation. Sample grouping and axis significance were analyzed by ANOVA. Samples with more than one pathogen were included in the analysis.

**Figure 5:**
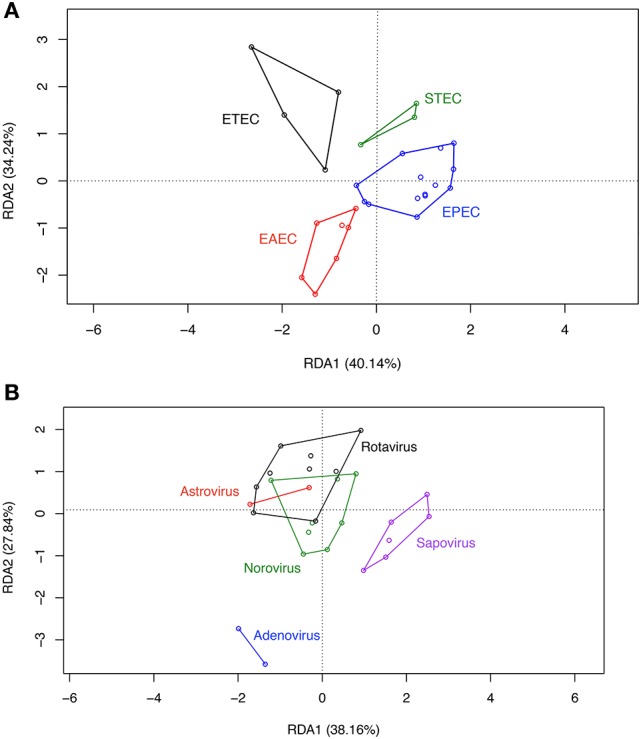
RDA on OPU data of DEC and viral group samples.Asymmetric correlation was done using sample classification as the explanatory matrix and the relative abundance of OPUS as the response matrix for DEC **(A)** or viral **(B)** samples. Data was normalized with a double square root transformation. Samples grouping and axis significance were analyzed by ANOVA. Samples with more than one pathogen were excluded from the analysis

RDA analysis of OPU composition for all groups revealed differences in community structure (Figure 4) which were statistically significant by ANOVA (*p* < 0.001). A separate preliminary RDA analysis showed that DEC pathotype aggregated in independent clusters (Figure 5A). This clustering was not observed when RDA was performed for diarrheal samples positive for enteric viral pathogens (Figure 5B).

### Indicative species

**Figure 6:**
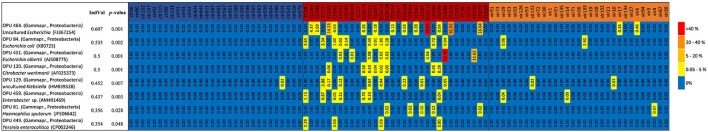
Heatmap of indicative species in the DEC group. Distribution of indicative species according their relative abundance in each sample. Healthy group (blue), DEC group (red), and viral group (green).

To identify indicative species for each group, we compared the OPU composition following De Cáceres and Legendre's protocol which analyzes abundance and relative presence of each OPU. We identified eight indicative OPUs for the DEC group compared to the viral and healthy groups, all of which belonged to phylum *Proteobacteria*. Figure 6 shows a heatmap of these indicative species, which were present in the majority of DEC positive diarrheal samples. A similar analysis was performed for the viral and healthy groups; this analysis identified 11 and 70 indicative species in each group, respectively (Supplementary Tables 2, 3).

## Discussion

Dysbiosis of the gut microbiota has been identified in patients with acute diarrhea, but association with specific pathogens is lacking. In this study, we found that stool samples of children positive for DEC pathotypes harbored a defined microbiota composition different to that found in children suffering an enteric virus associated diarrhea episode or in healthy children.

Our methodological approach was based on comparison of OPU composition differing from most studies which have been based on an OTU approach. The accuracy of OTUs for proper taxa assignment has been questioned (Guazzaroni et al., 2013) and in previous reports, the use of OPUs for taxa inference resulted in more reliable taxon assignment reducing overall diversity, providing clearer taxonomic data (Vidal et al., 2015). In our study, rarefaction curves showed that OTUs overestimated diversity while OPU based taxa assignment reached a maximum that was not influenced by adding more sequences (Figure 1). A high threshold for taxa assignment allowed us to identify 852 indisputable OPUs and manual review for identity between sequences, including the best available references (Yilmaz et al., 2014) and phylogenetic environment allow high confidence in assignments. The OPU approach allowed building a phylogenic tree for gastrointestinal microbiota from Chilean children. The tree was based on alignments with well-classified sequences, followed by taxon assignment by phylogenetic inference.

In contrast to other studies, ß-diversity was similar between groups (Hill and Artis, 2010; Pop et al., 2014). In our case, the similarity between ß-diversity indexes does not represent a similar diversity of groups. As previously postulated (Jost, 2006), true diversity cannot be explained by indexes, while a deep analysis of taxa abundance and richness may allow better understanding of group compositions. In our study, RDA analysis on OPUs allowed to show clear differences between groups, and identified a unique gut microbiota associated with DEC infections compared to enteric virus infections and healthy children. Some taxa were highly abundant and others were rare in DEC samples, probably due to different DEC pathotypes present in the stool samples. We normalized the data by double-square root transformation allowing the use of multivariate analysis based on correlation of data rather than raw distances. RDA and indicative taxa analysis confirmed a differential composition and the presence of indicative taxa for each etiological group (Figure 4). Although all DEC pathotypes share a common *E. coli* ancestor, the virulence mechanisms employed by each pathotype are different and depend on the expression of particular virulence genes (Kaper et al., 2004). RDA analysis in DEC group showed a clustering by pathotypes (Figure 5A); such clustering was not observed in the enteric viral group (Figure 5B). Due to low number of samples positives for each pathogen, it was not possible to perform statistical analyses. Further studies including more samples from each of the various DEC pathotypes would allow to determine the microbiota associated to each DEC pathotypes and define if these infections should be analyzed individually or as a group.

Among healthy children the gut microbiota has proven to be highly diverse during the early months of life. As age increases, microbial communities become more similar converging toward a generic adult-like profile, characterized by a preponderance of *Bacteroides* and *Firmicutes* and a low abundance of *Proteobacteria* (Palmer et al., 2007; Tojo et al., 2014). In diarrheal episodes, a change in phylum composition has been observed, with an increase in the abundance of *Proteobacteria* and *Bacteroidetes* and a decrease in *Firmicutes* (Braun et al., 2017). Our results are in agreement with these previous reports, as we found high proportion of *Bacteroidetes* and *Proteobacteria* in the DEC group compared to healthy group (Figure 2A). The low abundance of *Firmicutes* in diarrheal group could have an important impact in this children. Metatranscriptomic studies have shown that *Lachnospiraceae* and *Ruminococcaceae* families belonging to *Firmicutes phylum*, play an important role in dietary fibers metabolism. Likewise, *Ruminococcaceae* has been associated to antibiotic biosynthesis, important on host defense against pathogens (Gosalbes et al., 2011).

At the family level, a loss of diversity in the intestinal microbiota is observed during intestinal infections caused by diverse pathogens, with an increased abundance of *Enterobacteriaceae* compared to healthy subjects (Stecher et al., 2007; Hill and Artis, 2010; Arumugam et al., 2011). Concordant with these observations, in our DEC group all indicative taxa belong to the *Enterobacteriaceae* family. The presence of the genera *Citrobacter, Enterobacter*, and *Haemophilus* are consistent with previous observations (Pop et al., 2014). The abundance of *Escherichia/Shigella* species in the DEC group compared to viral and healthy groups might be attributable to the presence of DEC pathotype in the diarrheal samples.

The majority of the indicative species in the DEC group identified in this study have been previously associated with diarrheal episodes. *Escherichia albertii* is an emerging human enteropathogen and avian pathogen (Huys et al., 2003; Ooka et al., 2015), that shares the ability of EPEC and STEC to promote attaching-effacing (A/E) lesions due to the presence of locus of enterocyte effacement (LEE) genes (Yamamoto et al., 2017). *Yersinia enterocolitica* is a well-known gastrointestinal pathogen that causes yersiniosis, an illness characterized by diarrhea, ileitis, and mesenteric lymphadenitis (Gupta et al., 2015). Some species of the genus *Klebsiella* have been associated with diarrheal disease, for example, *Klebsiella oxytoca* is one of the ethological microorganism of antibiotic-associated hemorrhagic colitis (Zollner-Schwetz et al., 2008). It is important to note, that our analysis could not distinguished between pathogenic or commensal bacteria belonging to the same species. Previous reports have shown that components of the gut microbiota impact the expression of virulence factors. *Bacteroides tethaiotaomicron*, a predominant member of the gut microbiota in healthy subjects, enhanced the expression of several genes of STEC reference strain in co-culture experiments, including virulence genes located in the LEE pathogenicity island. Moreover, an increase of A/E lesions and STEC adherence to epithelial cells was found (Curtis et al., 2014; Iversen et al., 2015). Considering the above, further studies should focus on the role of species described here as indicative of DEC group in the pathogenicity of DEC pathotypes.

One of the strengths of our study is the construction of a phylogenetic tree. To our knowledge, this is the first phylogenetic tree built from reference sequences to study the gut microbiota based on OPU identification in diarrheal and non-diarrheal stool samples of children. This tree was manually supervised and can serve as a tool to characterize the taxa composition not only in samples associated with diarrhea, but also other gastrointestinal diseases. We used FilmArray® GI testing for pathogen detection; this diagnostic tool that has been increasingly used for detection of enteric pathogens, as it provides rapid, sensitive results for the most relevant enteric pathogens including bacteria, viruses and parasites. It is important to note, that FilmArray® GI testing is FDA-approved exclusively for diarrheal stool samples, and its use in stool samples from healthy individuals has not been validated.

Our study has limitations. Inclusion criteria were children aged 1–5 years with an episode of diarrhea. It is well known that several environmental and host factors, such as, age, food consumption, economic status, antibiotic treatment, and others might influence the gut microbiota composition (Sartor, 2012; Bäumler and Sperandio, 2016). Our healthy children were from the city of Colina, a middle-low income town in the outskirts of Santiago while the diarrhea cases were children consulting in a private clinic within the high socioeconomic sector of Santiago. Lack of matching for the other variables may also be playing a role in microbiome differences between groups. Even though we did not find significant difference in age between groups, the mean age of children in DEC group was lower than the other groups. Considering that variability of gut microbiota in children <3 years is high, age-matched studies with stricter inclusion and exclusion criteria are necessary to validated our findings.

In conclusion, we found a distinctive gut microbiota that is associated with DEC infections compared to enteric virus infections and healthy children. The identification of indicative species in the DEC group are relevant in advancing our understanding as to the involvement of the gut microbiota in DEC pathogenicity.

## Author contributions

PG and MI processed the samples, performed sequence analysis, interpreted the data, and participated in manuscript writing. MF participated in study design, acquisition of data, interpretation of data, manuscript writing, and final approval of the manuscript. NC participated in data analysis. RR supervised our OPU approach, reviewed taxonomic inference, and helped with data analysis. RV participated in study design and data analysis. MO participated in the study design and data analysis.

### Conflict of interest statement

The authors declare that the research was conducted in the absence of any commercial or financial relationships that could be construed as a potential conflict of interest.

## Supplementary material

Supplementary Figure 1 Phyla distribution on NJ_30 tree. Three main phyla are highlighted and location of other phyla indicated on dash lines. Indicative taxa of DEC group appear on red lines within *Proteobacteria*. A close up of OPU 120 is shown. Reference sequence on green and aligned sample sequences on red sharing a monophyletic origin.

Supplementary Figure 2 Sørensen ß-diversity. Centroids show the median Sørensen index value for each sample. No significant differences (*p* = 0.080).

Supplementary Figure 3 Heatmap of identified species. Distribution of indicative species according their relative abundance in each sample. Healthy group (blue), DEC group (red), and viral group (green).
