# Supplementary material for: Distinctive Gut Microbiota Is Associated with Diarrheagenic Escherichia coli Infections in Chilean Children
Source: Front Cell Infect Microbiol. 2017 Oct 12;7:424. doi: 10.3389/fcimb.2017.00424 (PMC5643428; doi:10.3389/fcimb.2017.00424)
Supplement: Supplementary Figure 1 — Phyla distribution on NJ_30 tree. Three main phyla are highlighted and location of other phyla indicated on dash lines. Indicative taxa of DEC group appear on red lines within Proteobacteria. A close up of OPU 120 is shown. Reference sequence on green and aligned sample sequences on red sharing a monophyletic origin. [file DataSheet1.PDF]

Supplementary Figure 1

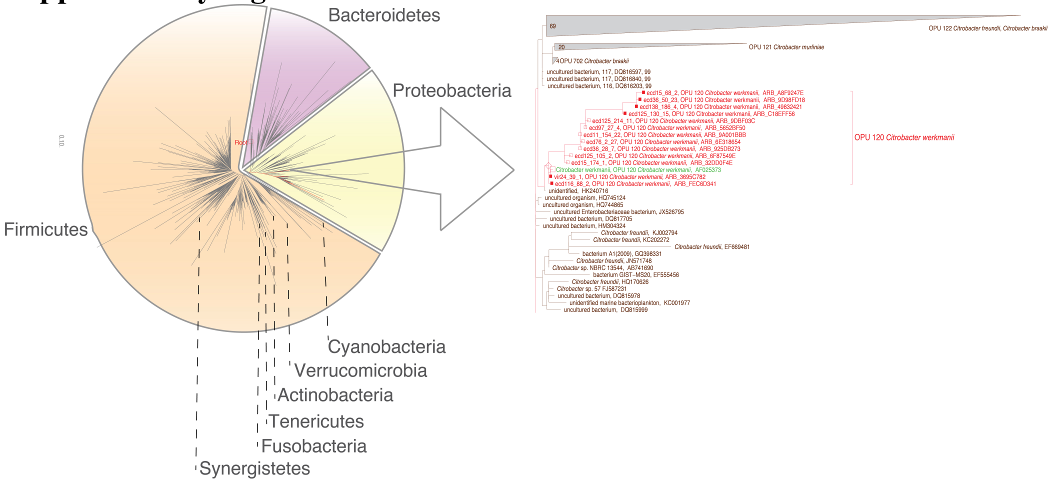

Supplementary Figure 2.

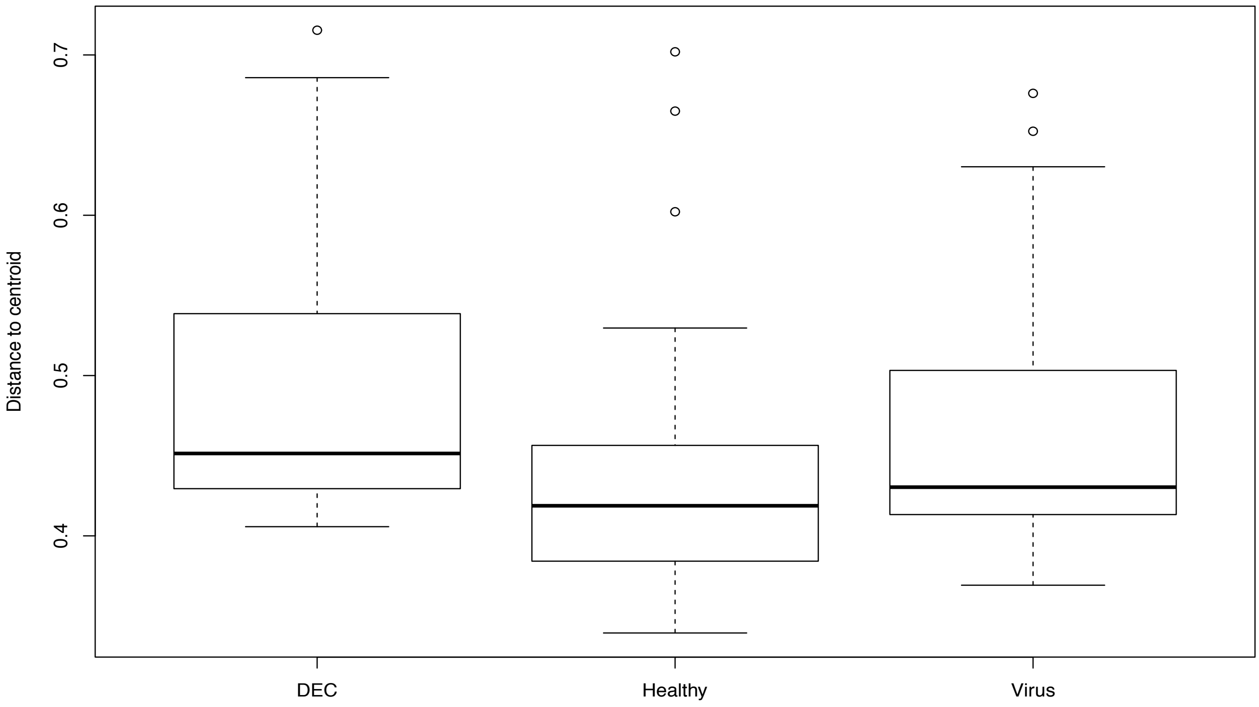

Supplementary Figure 3.

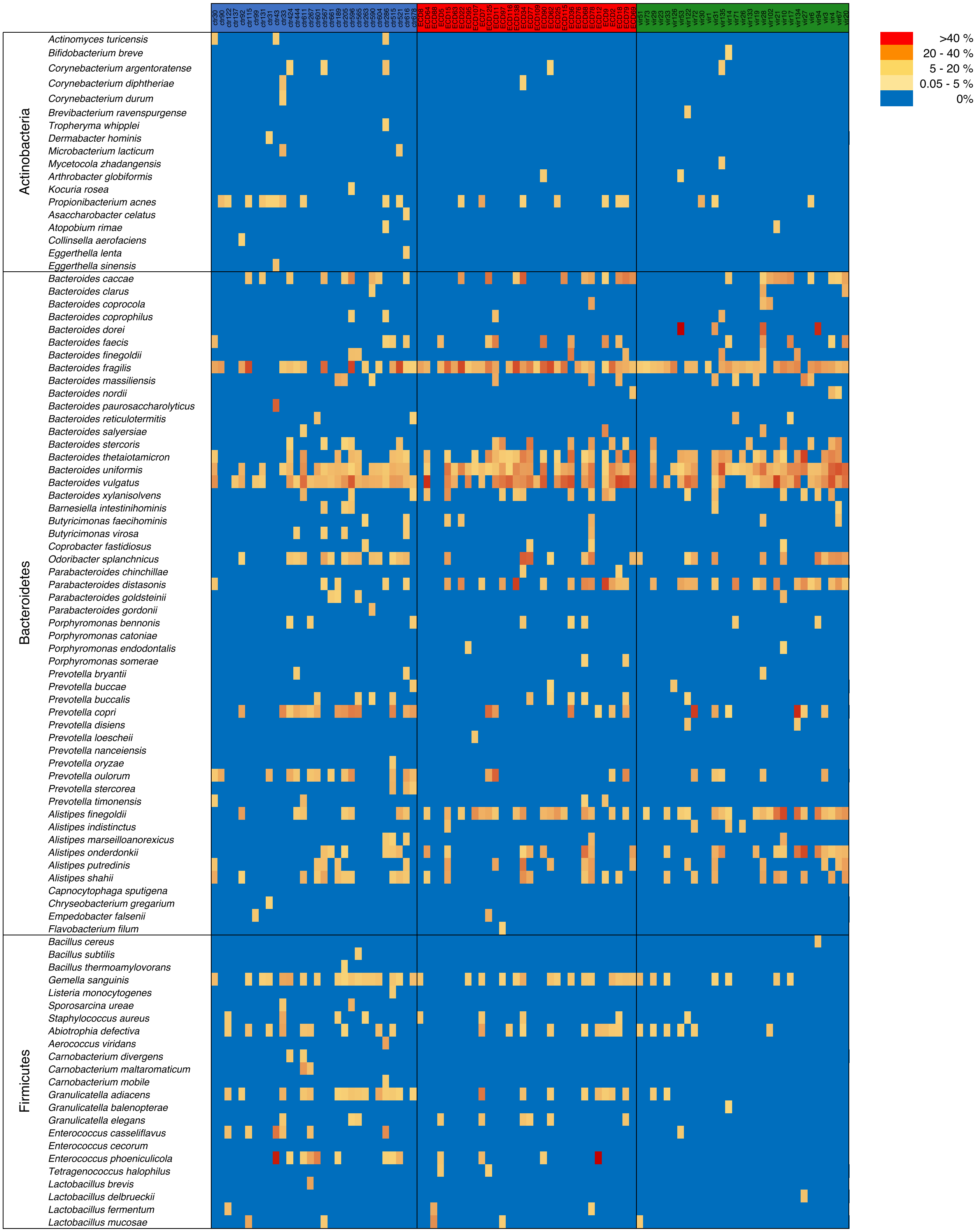

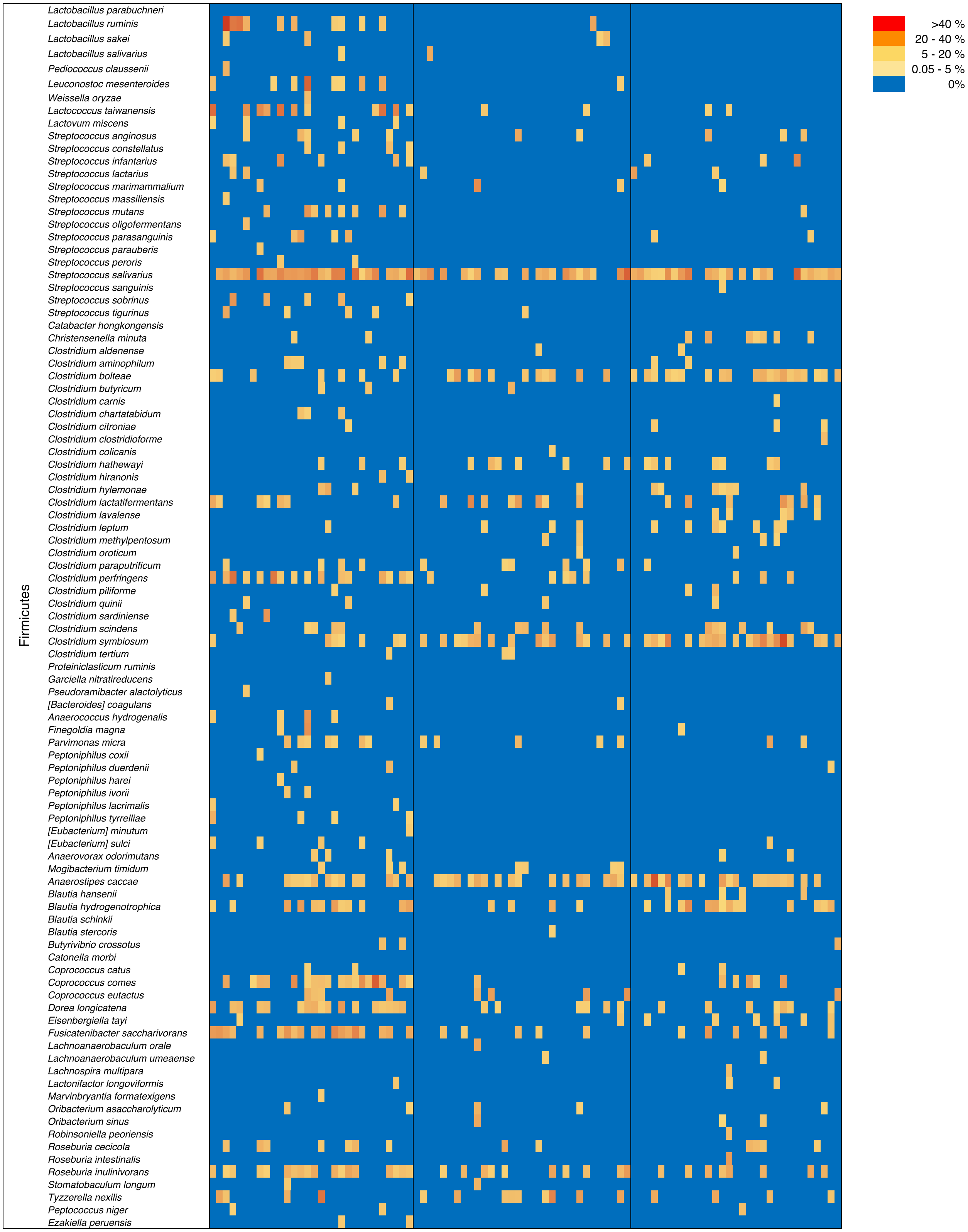
Firmicutes
Lactobacillus parabuchneri
Lactobacillus ruminis
Lactobacillus sakei
Lactobacillus salivarius
Pediococcus claussenii
Leuconostoc mesenteroides
Weissella oryzae
Lactococcus taiwanensis
Lactovum miscens
Streptococcus anginosus
Streptococcus constellatus
Streptococcus infantarius
Streptococcus lactarius
Streptococcus marimammalium
Streptococcus massiliensis
Streptococcus mutans
Streptococcus oligofermentans
Streptococcus parasanguinis
Streptococcus parauberis
Streptococcus peroris
Streptococcus salivarius
Streptococcus sanguinis
Streptococcus sobrinus
Streptococcus tigurinus
Catabacter hongkongensis
Christensenella minuta
Clostridium aldenense
Clostridium aminophilum
Clostridium bolteae
Clostridium butyricum
Clostridium carnis
Clostridium chartatabidum
Clostridium citroniae
Clostridium clostridioforme
Clostridium colicanis
Clostridium hathewayi
Clostridium hiranonis
Clostridium hylemonae
Clostridium lactatifermentans
Clostridium lavalense
Clostridium leptum
Clostridium methylpentosum
Clostridium oroticum
Clostridium paraputrificum
Clostridium perfringens
Clostridium piliforme
Clostridium quinii
Clostridium sardiniense
Clostridium scindens
Clostridium symbiosum
Clostridium tertium
Proteiniclastium ruminis
Garciella nitratireducens
Pseudoramibacter alactolyticus
[Bacteroides] coagulans
Anaerococcus hydrogenalis
Finegoldia magna
Parvimonas micra
Peptoniphilus coxii
Peptoniphilus duerdenii
Peptoniphilus harei
Peptoniphilus ivorii
Peptoniphilus lacrimalis
Peptoniphilus tyrrelliae
[Eubacterium] minutum
[Eubacterium] sulci
Anaerovorax odorimutans
Mogibacterium timidum
Anaerostipes caccae
Blautia hansenii
Blautia hydrogenotrophica
Blautia schinkii
Blautia stercoris
Butyrivibrio crossotus
Catonella morbi
Coprococcus catus
Coprococcus comes
Coprococcus eutactus
Dorea longicatena
Eisenbergiella tayi
Fusicatenibacter saccharivorans
Lachnoanaerobaculum orale
Lachnoanaerobaculum umeaense
Lachnospira multipara
Lactonifactor longoviformis
Marvinbryantia formatexigens
Oribacterium asaccharolyticum
Oribacterium sinus
Robinsoniella peoriensis
Roseburia cecicola
Roseburia intestinalis
Roseburia inulinivorans
Stomatobaculum longum
Tyzzerella nexilis
Peptococcus niger
Ezakiella peruensis
>40 %
20 - 40 %
5 - 20 %
0.05 - 5 %
0%

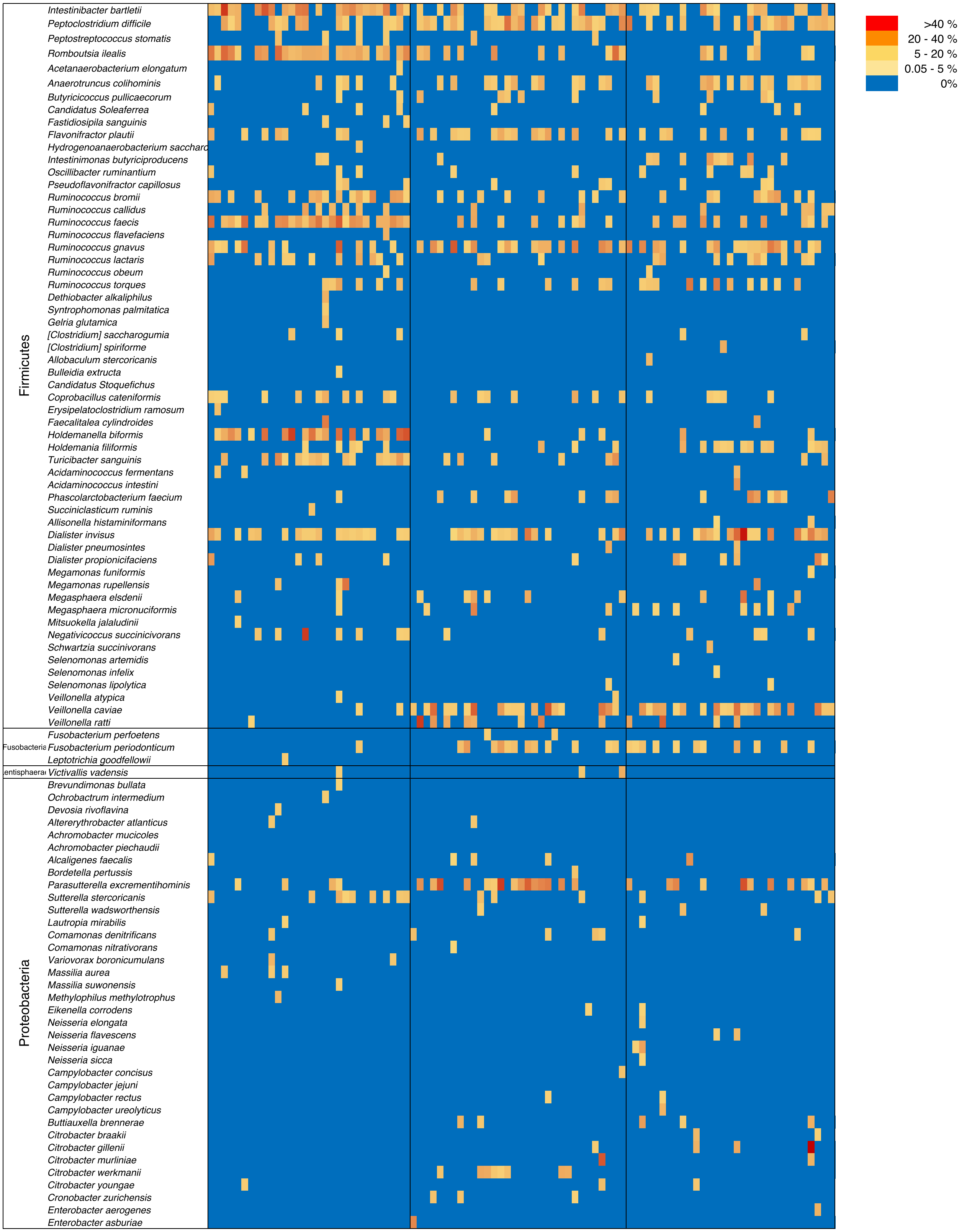

Firmicutes
Intestinibacter bartletii
Peptoclostridium difficile
Peptostreptococcus stomatis
Romboutsia ilealis
Acetanaerobacterium elongatum
Anaerotruncus colihominis
Butyricicoccus pullicaecorum
Candidatus Soleaferrea
Fastidiosipila sanguinis
Flavonifractor plautii
Hydrogenoanaerobacterium saccharo
Intestinimonas butyriciproducens
Oscillibacter ruminantium
Pseudoflavonifractor capillosus
Ruminococcus bromii
Ruminococcus callidus
Ruminococcus faecis
Ruminococcus flavefaciens
Ruminococcus gnavus
Ruminococcus lactaris
Ruminococcus obeum
Ruminococcus torques
Dethiobacter alkaliphilus
Syntrophomonas palmitatica
Gelria glutamica
[Clostridium] saccharogumia
[Clostridium] spiriforme
Allobaculum stercoricanis
Bulleidia extructa
Candidatus Stoquefichus
Coprobacillus cateniformis
Erysipelatoclostridium ramosum
Faecalitalea cylindroides
Holdemanella biformis
Holdemania filiformis
Turicibacter sanguinis
Acidaminococcus fermentans
Acidaminococcus intestini
Phascolarctobacterium faecium
Succiniclasticum ruminis
Allisonella histaminiformans
Dialister invisus
Dialister pneumosintes
Dialister propionicifaciens
Megamonas funiformis
Megamonas rupellensis
Megasphaera elsdenii
Megasphaera micronuciformis
Mitsuokella jalaludinii
Negativicoccus succinicivorans
Schwartzia succinivorans
Selenomonas artemidis
Selenomonas infelix
Selenomonas lipolytica
Veillonella atypica
Veillonella caviae
Veillonella ratti
Fusobacteria
Fusobacterium perfoetens
Fusobacterium periodonticum
Leptotrichia goodfellowii
entisphaera
Victivallis vadensis
Proteobacteria
Brevundimonas bullata
Ochrobactrum intermedium
Devosia rivoflavina
Altererythrobacter atlanticus
Achromobacter mucicoles
Achromobacter piechaudii
Alcaligenes faecalis
Bordetella pertussis
Parasutterella excrementihominis
Sutterella stercoricanis
Sutterella wadsworthensis
Lautropia mirabilis
Comamonas denitrificans
Comamonas nitrativorans
Variovorax boronicumulans
Massilia aurea
Massilia suwonensis
Methylophilus methylotrophus
Eikenella corrodens
Neisseria elongata
Neisseria flavescens
Neisseria iguanae
Neisseria sicca
Campylobacter concisus
Campylobacter jejuni
Campylobacter rectus
Campylobacter ureolyticus
Buttiauxella brennerae
Citrobacter braakii
Citrobacter gillenii
Citrobacter murliniae
Citrobacter werkmanii
Citrobacter youngae
Cronobacter zurichensis
Enterobacter aerogenes
Enterobacter asburiae
>40 %
20 - 40 %
5 - 20 %
0.05 - 5 %
0%

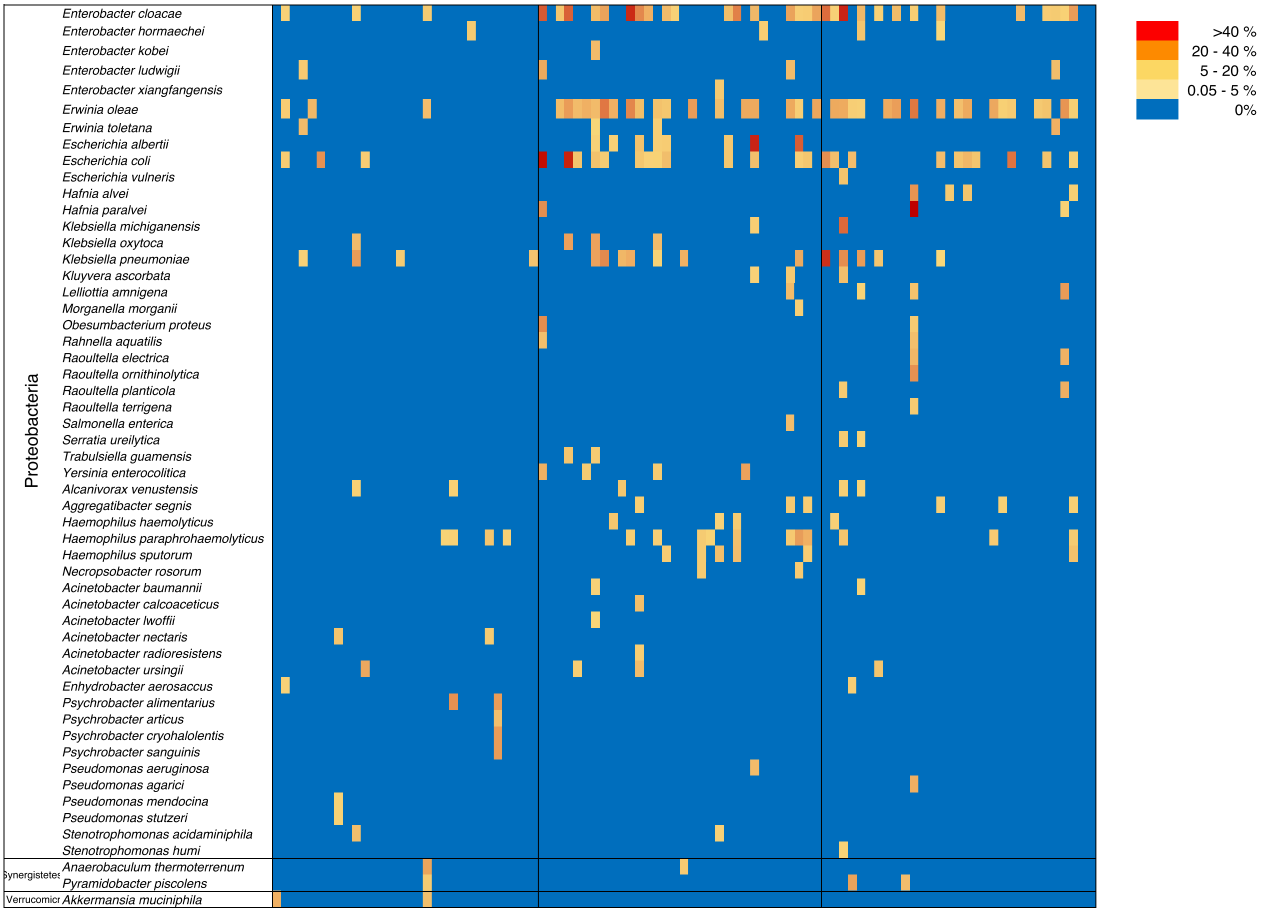

Proteobacteria
Synergistetes
Verrucomicr
Enterobacter cloacae
Enterobacter hormaechei
Enterobacter kobei
Enterobacter ludwigii
Enterobacter xiangfangensis
Erwinia oleae
Erwinia toletana
Escherichia albertii
Escherichia coli
Escherichia vulneris
Hafnia alvei
Hafnia paralvei
Klebsiella michiganensis
Klebsiella oxytoca
Klebsiella pneumoniae
Kluyvera ascorbata
Lelliottia amnigena
Morganella morganii
Obesumbacterium proteus
Rahnella aquatilis
Raoultella electrica
Raoultella ornithinolytica
Raoultella planticola
Raoultella terrigena
Salmonella enterica
Serratia ureilytica
Trabulsiella guamensis
Yersinia enterocolitica
Alcanivorax venustensis
Aggregatibacter segnis
Haemophilus haemolyticus
Haemophilus paraphrohaemolyticus
Haemophilus sputorum
Necropsobacter rosorum
Acinetobacter baumannii
Acinetobacter calcoaceticus
Acinetobacter lwoffii
Acinetobacter nectaris
Acinetobacter radioresistens
Acinetobacter ursingii
Enhydrobacter aerosaccus
Psychrobacter alimentarius
Psychrobacter articus
Psychrobacter cryohalolentis
Psychrobacter sanguinis
Pseudomonas aeruginosa
Pseudomonas agarici
Pseudomonas mendocina
Pseudomonas stutzeri
Stenotrophomonas acidaminiphila
Stenotrophomonas humi
Anaerobaculum thermoterrenum
Pyramidobacter piscolens
Akkermansia muciniphila
>40 %
20 - 40 %
5 - 20 %
0.05 - 5 %
0%

Supplementary Table 1: Primers

rDNA 16S primers	
**GM3**	AGAGTTTGATCMTGGC (G. Muyzer y cols., 1993)
**1492R**	GGTTACCTTGTTACGACTT (Weisburg, Barns, Pelletier, & Lane, 1991)
**Primers 454® - pyrosequencing**	
**Name**	**5' - 3' sequence**
GM3-PS	Adapter B-TCAG-AGAGTTTGATCMTGGC
907-PS R1	Adapter A-TCAG-ACGAGTGCGT CCGTCAATTCMTTTGAGTTT
907-PS R2	Adapter A-TCAG-ACGCTCGACA CCGTCAATTCMTTTGAGTTT
907-PS R3	Adapter A-TCAG-AGACGCACTC CCGTCAATTCMTTTGAGTTT
907-PS R4	Adapter A-TCAG-AGCACTGTAG CCGTCAATTCMTTTGAGTTT
907-PS R5	Adapter A-TCAG-ATCAGACACG CCGTCAATTCMTTTGAGTTT
907-PS R6	Adapter A-TCAG-ATATCGCGAG CCGTCAATTCMTTTGAGTTT
907-PS R7	Adapter A-TCAG-CGTGTCTCTA CCGTCAATTCMTTTGAGTTT
907-PS R8	Adapter A-TCAG-CTCGCGTGTC CCGTCAATTCMTTTGAGTTT
907-PS R9	Adapter A-TCAG-TAGTATCAGC CCGTCAATTCMTTTGAGTTT
907-PS R10	Adapter A-TCAG-TCTCTATGCG CCGTCAATTCMTTTGAGTTT
907-PS R11	Adapter A-TCAG-TGATACGTCT CCGTCAATTCMTTTGAGTTT
907-PS R12	Adapter A-TCAG-TACTGAGCTA CCGTCAATTCMTTTGAGTTT
907-PS R13	Adapter A-TCAG-CATAGTAGTG CCGTCAATTCMTTTGAGTTT
907-PS R14	Adapter A-TCAG-CGAGAGATAC CCGTCAATTCMTTTGAGTTT
907-PS R15	Adapter A-TCAG-ATACGACGTA CCGTCAATTCMTTTGAGTTT
907-PS R16	Adapter A-TCAG-TCACGTACTA CCGTCAATTCMTTTGAGTTT
907-PS R17	Adapter A-TCAG-CGTCTAGTAC CCGTCAATTCMTTTGAGTTT
907-PS R18	Adapter A-TCAG-TCTACGTAGC CCGTCAATTCMTTTGAGTTT
907-PS R19	Adapter A-TCAG-TGTACTACTC CCGTCAATTCMTTTGAGTTT
907-PS R20	Adapter A-TCAG-ACGACTACAG CCGTCAATTCMTTTGAGTTT
907-PS R21	Adapter A-TCAG-CGTAGACTAG CCGTCAATTCMTTTGAGTTT
907-PS R22	Adapter A-TCAG-TACGAGTATG CCGTCAATTCMTTTGAGTTT
907-PS R23	Adapter A-TCAG-TACTCTCGTG CCGTCAATTCMTTTGAGTTT
Adapter B: CCTATCCCCTGTGTGCCTTGGCAGTC	
Adapter A: CCATCTCATCCCTGCGTGTCTCCGAC	
Gauge: TCAG	
Barcode sequence	
rDNA 16S primer: CCGTCAATTCMTTTGAGTTT (Gerard Muyzer & Smalla, 1998)	

Supplementary Table 2: Indicative taxas for Viral group.

OPU #	Closest specie	Access number	stat	*p*-value
171	*Holdemania filiformis*	Y11466	0.495	0.041
237	*Intestimonas butyriciproducens*	KC311367	0.487	0.002
229	*Christensenella minuta*	AB490809	0.433	0.005
468	uncultured Verrucomicrobiaceae	X90515	0.432	0.018
250	*[Clostridium] lavalense*	EF564277	0.402	0.003
252	*Eisenbergiella tayi*	KF814111	0.384	0.016
613	uncultured *Subdoligranulum*	DQ825098	0.378	0.030
503	*Blautia hansenii*	AB534168	0.359	0.024
765	*Hafnia alvei*	M59155	0.359	0.020
770	*Bacteroides dorei*	AB242142	0.359	0.027

Supplementary Table 3: Indicative taxas for Healthy group.

OPU #	Closest species	Access number	stat	*p*-value
395	uncultured Erysipelotrichaceae UCG-003	BAAU01005302	0.774	0.001
534	[Eubacterium] hallii Group	DQ795004	0.770	0.001
720	Ruminococcus faecis	FJ611794	0.762	0.001
172	Holdemanella biformis	M59230	0.752	0.001
300	uncultured Clostridium senso stricto	DQ801127	0.734	0.001
318	Romboutsia ilealis	JN381505	0.729	0.001
612	uncultured *Subdoligranulum*	DQ802292	0.711	0.001
279	*Coprococcus comes*	EF031542	0.675	0.001
254	*Fusicatenibacter saccharivorans*	AB698910	0.675	0.001
373	uncultured Christensenellaceae R-7 group	AB189701	0.646	0.001
587	*Clostridium perfringens*	AB910734	0.633	0.001
481	uncultured Christensenellaceae R-7 group	EU779015	0.620	0.001
297	*Dorea longicatena*	AJ132842	0.607	0.006
168	uncultured Mollicutes	AY239433	0.600	0.001
166	*Turicibacter sanguinis*	AF349724	0.595	0.001
724	uncultured Lachnospiraceae	AB506222	0.581	0.001
202	*Lactococcus taiwanensis*	AB699722	0.572	0.001
631	uncultured *Intestinibacter*	EU775215	0.556	0.010
195	*Lactobacillus ruminis*	AB326354	0.553	0.001
599	uncultured *Coprococcus*	AJ270485	0.524	0.004
667	uncultured Ruminococcaceae NK4A214	AB218309	0.523	0.002
639	Christensenellaceae R-7 group	DQ809041	0.519	0.001
176	*Catenibacterium mitsuokai*	AB030224	0.508	0.001
715	uncultured *Lactococcus*	AB234513	0.506	0.004
346	uncultured *Phascolarctobacterium* / *Phascolarctobacterium succinatutens*	AB490811	0.500	0.003
184	*Leuconostoc mesenteroides* subsp. *mesenteroides*	CP000414	0.500	0.001
307	[Eubacterium] eligens	L34420	0.499	0.022
205	*Streptococcus mutans*	AY188348	0.496	0.001
308	*Bacteroides galacturonicus / Lactobacillus rogosae*	DQ497994 / GU269544	0.493	0.048
535	uncultured Lachnospiraceae NK4A136 Group	FJ504036	0.488	0.001
575	uncultured Roseburia	AY269188	0.487	0.007
669	uncultured Ruminococcaceae UCG-010	AF050581	0.485	0.002
349	uncultured Coriobacteriaceae	AB192289	0.474	0.004
341	*Sutterella stercoricanis*	AJ566849	0.438	0.050
662	uncultured Ruminococcaceae UCG-010	AB506250	0.431	0.034
200	*Enterococcus casseliflavus*	AF039903	0.429	0.002
160	*Actinomyces* sp.	JRMV01000269	0.423	0.021
527	uncultured *Marvinbryantia*	DQ327473	0.421	0.006
494	uncultured Lachnospiraceae NC-2004 group	DQ807845	0.415	0.007
497	uncultured Lachnospiraceae NK4A136 group	EU764230	0.415	0.004
347	uncultured *Dialister*	KF843481	0.410	0.018
206	*Streptococcus sobrinus*	AY188349	0.408	0.006
603	uncultured Lachnospiraceae ND 3007	GQ896713	0.407	0.008

542	uncultured *Blautia*	DQ807838	0.392	0.019
11	uncultured Bacteroidales S24-7 Group	EU772376	0.382	0.016
133	uncultured *Vampirovibrio*	AM500751	0.365	0.008
218	*Peptoniphilus tyrrelliae*	GU938835	0.365	0.007
276	*Lactococcus piscium / Lactococcus plantarum*	DQ343754 / EF694929	0.365	0.009
469	uncultured Coriobacteriaceae	DQ800846	0.365	0.011
556	uncultured Lachnospiraceae	DQ803378	0.365	0.014
685	*Anaerococcus hydrogenalis*	ABXA01000039	0.365	0.017
709	*Lactovum miscens*	AJ439543	0.365	0.008
712	*Streptococcus constellatus*	JN787160 / AY309095	0.365	0.009
518	uncultured *Lachnospiraceae*	FJ504169	0.364	0.028
364	*Streptococcus parasanguinis*	AF003933	0.364	0.032
502	uncultured *Blautia*	DQ800033	0.363	0.023
545	uncultured *Anaerovorax* sp	KC894538	0.355	0.019
532	[Clostridium] *aminophilum*	L04165	0.351	0.030
478	uncultured Ruminococcaceae UCG-014 group	FJ362645	0.337	0.029
756	*Streptococcus tigurinus*	JN004270	0.334	0.023
362	*Lactobacillus plantarum / Lactobacillus pentosus*	AJ640078 / D79211	0.327	0.041
375	[Eubacterium] *sulci*	AJ006963	0.324	0.050
27	*Prevotella stercorea*	AB244774	0.316	0.032
75	*Massilia aurea*	AM231588	0.316	0.021
159	*Actinomyces turicensis*	X78720	0.316	0.026
201	*Lactococcus formosensis / Lactococcus garvieae*	AB775178 / AB598994	0.316	0.037
303	*Clostridium chartatabidum*	X71850	0.316	0.041
368	*Murdochiella asaccharolytica*	EU483153	0.316	0.024
500	uncultured *Blautia*	DQ798057	0.316	0.022
537	uncultured Lachnospiraceae	EF398740	0.316	0.032
544	uncultured Lachnoclostridium	DQ795955	0.316	0.029
734	uncultured Lachnospiraceae UCG-001	EF100046	0.316	0.031
787	*Fastidiosipila sanguinis*	AJ575187	0.316	0.026
842	uncultured Bacteroidales	GQ358389	0.316	0.036
385	uncultured Ruminococcaceae UCG-009	EU506312	0.284	0.050
